# Supplementary material for: Hydrodynamic tearing of bacteria on nanotips for sustainable water disinfection
Source: Nat Commun. 2023 Sep 15;14:5734. doi: 10.1038/s41467-023-41490-5 (PMC10504294; doi:10.1038/s41467-023-41490-5)
Supplement: Supplementary file 1 — Supplementary Information [file 41467_2023_41490_MOESM1_ESM.pdf]

Supplementary Information for

Hydrodynamic tearing of bacteria on nanotips for sustainable water disinfection

Lu Peng[1]†, Haojie Zhu[2]†, Haobin Wang[3], Zhenbin Guo[2,4], Qianyuan Wu[1]*, Cheng Yang[2]*, Hong-Ying Hu[1,3]*

Correspondence to: hyhu@tsinghua.edu.cn; yang.cheng@sz.tsinghua.edu.cn; wu.qianyuan@sz.tsinghua.edu.cn

This PDF file includes:

Supplementary Figs. 1–25
Supplementary Tables 1–7
Supplementary References

Other Supplementary Information include the following:

Supplementary Movies 1–11

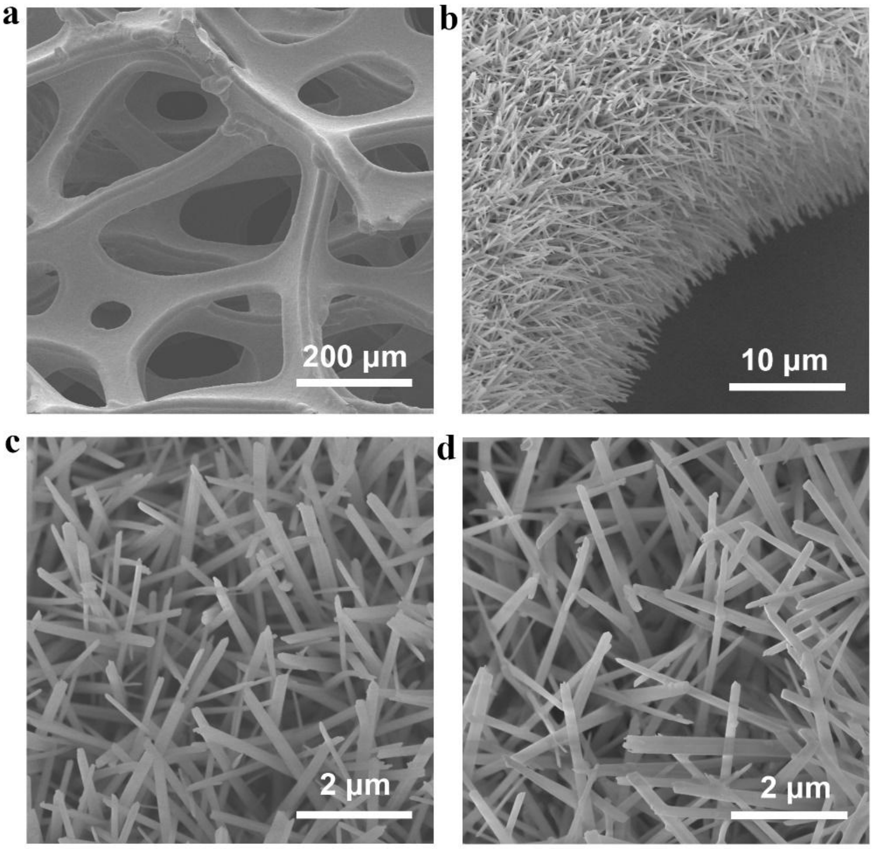

Supplementary Fig. 1 | Morphologies of the copper foam, $Cu(OH)_2$ NWs and modified NWs. a, SEM image of copper foam. **b**,**c** Low- and high- magnification SEM images of the $Cu(OH)_2$ NWs. **d**, SEM image of the modified NWs. Observations were repeated four times independently with similar results.

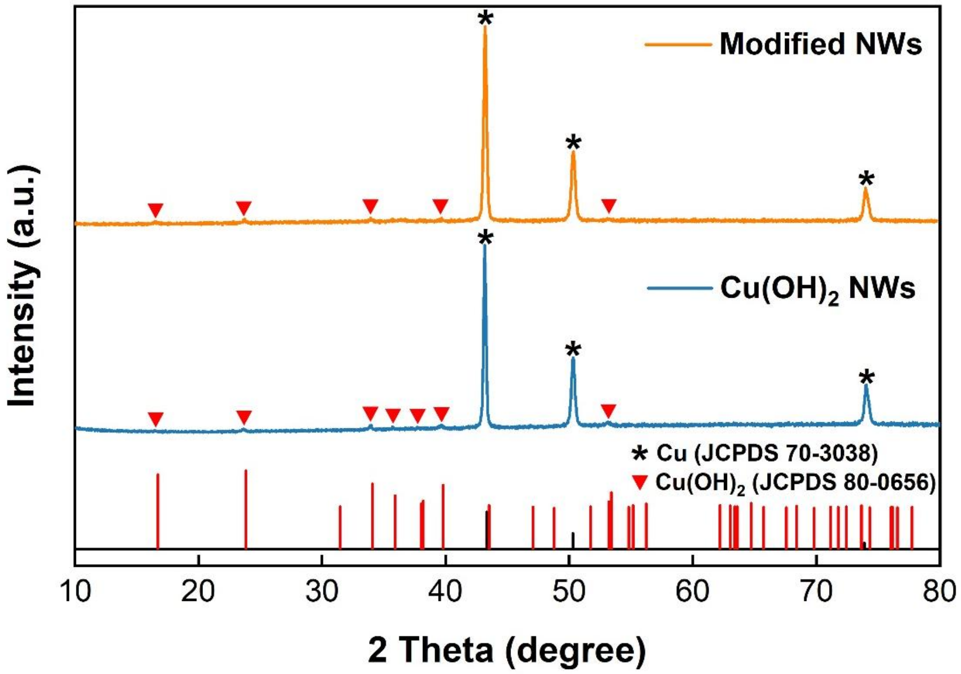

Supplementary Fig. 2 | XRD patterns of the $Cu(OH)_2$ NWs and the modified NWs. Source data are provided as a Source Data file.

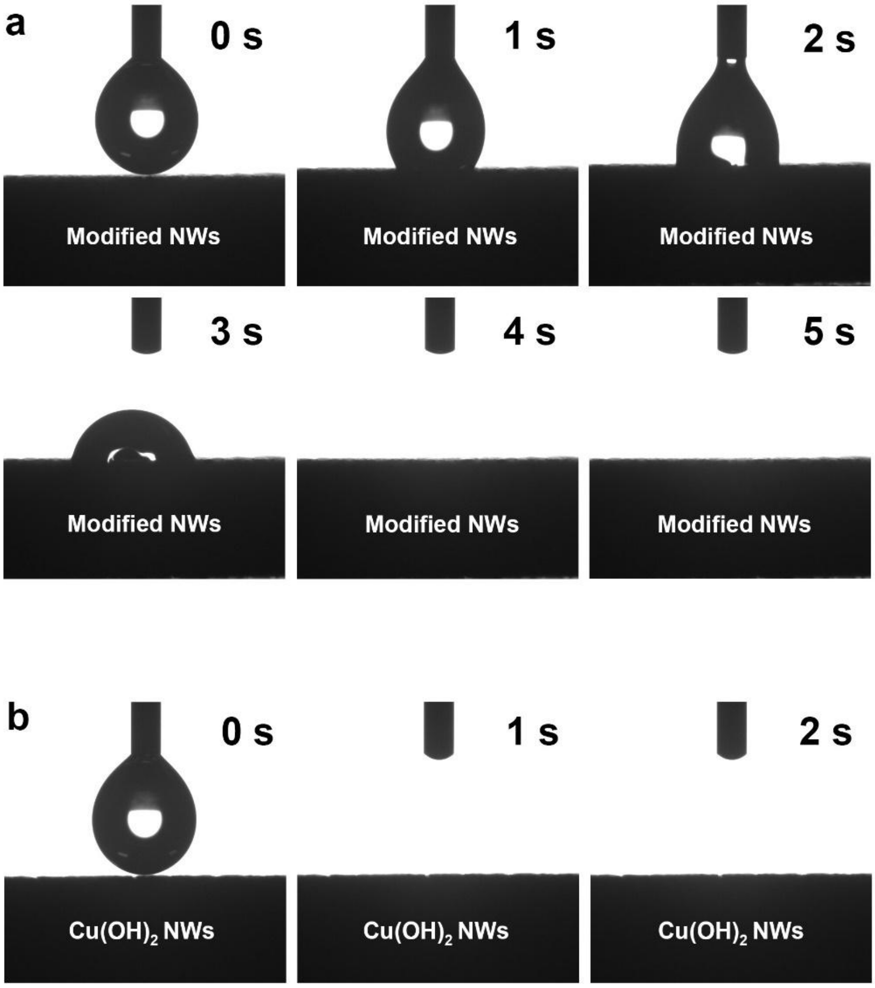

Supplementary Fig. 3 | Water contact angle measurements. **a**, Modified NWs. **b**, $Cu(OH)_2$ NWs.

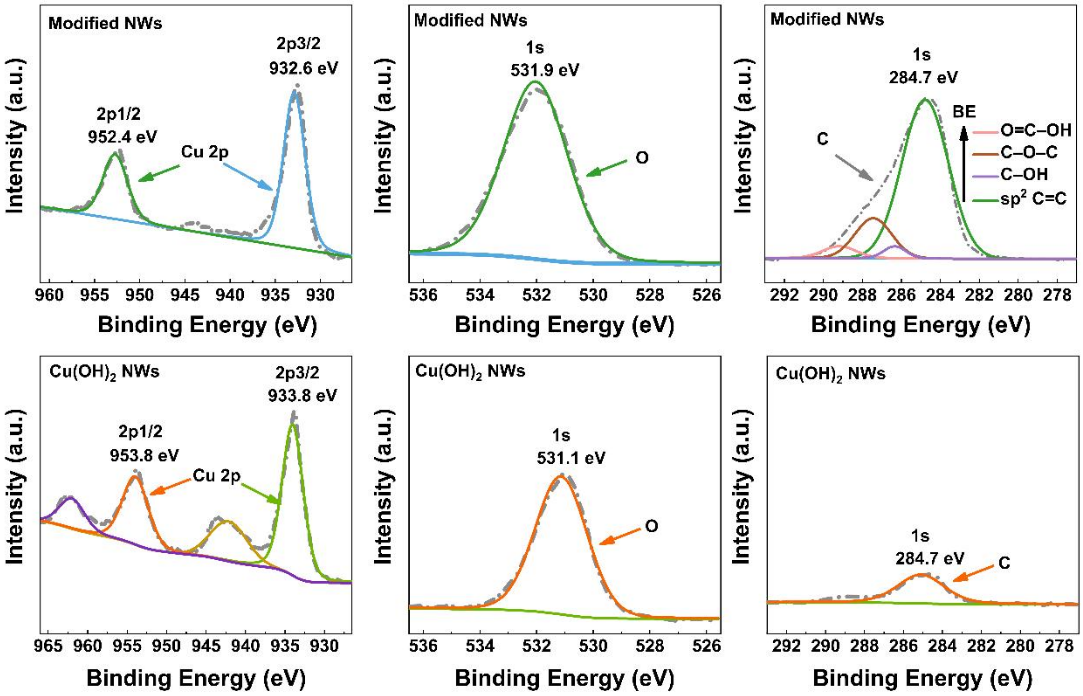

Supplementary Fig. 4 | XPS spectra of the $Cu(OH)_2$ NWs and the modified NWs. Source data are provided as a Source Data file.

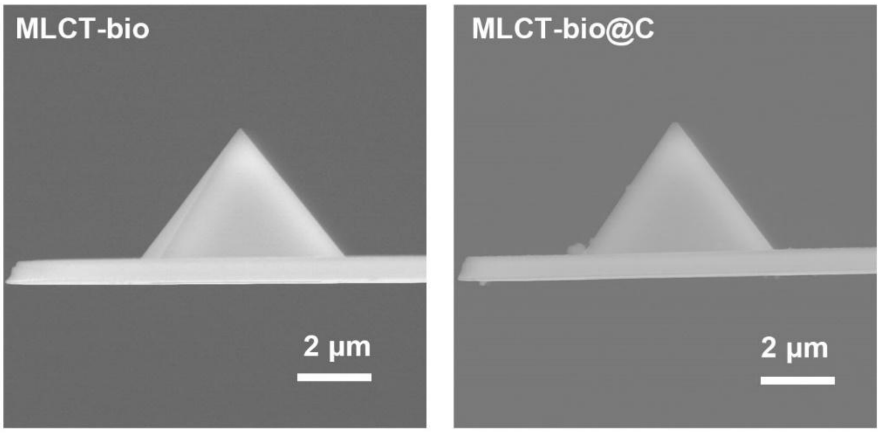

Supplementary Fig. 5 | Morphologies of the original and carbon-coated AFM tips. Observations were repeated three times independently with similar results.

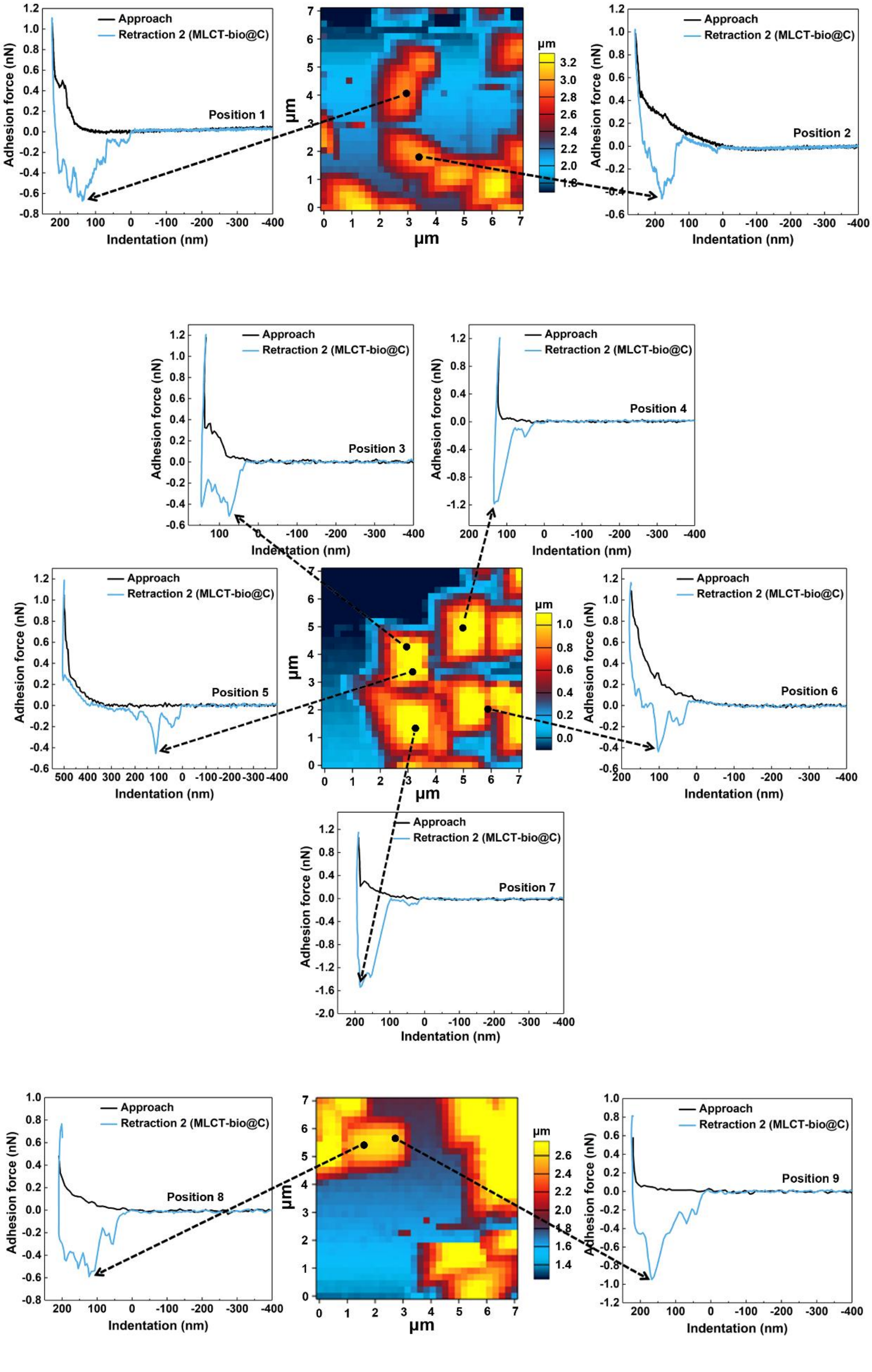

Supplementary Fig. 6 | Adhesion force between an *E. coli* cell and a carbon-coated AFM tip (positions 1–9). Source data are provided as a Source Data file.

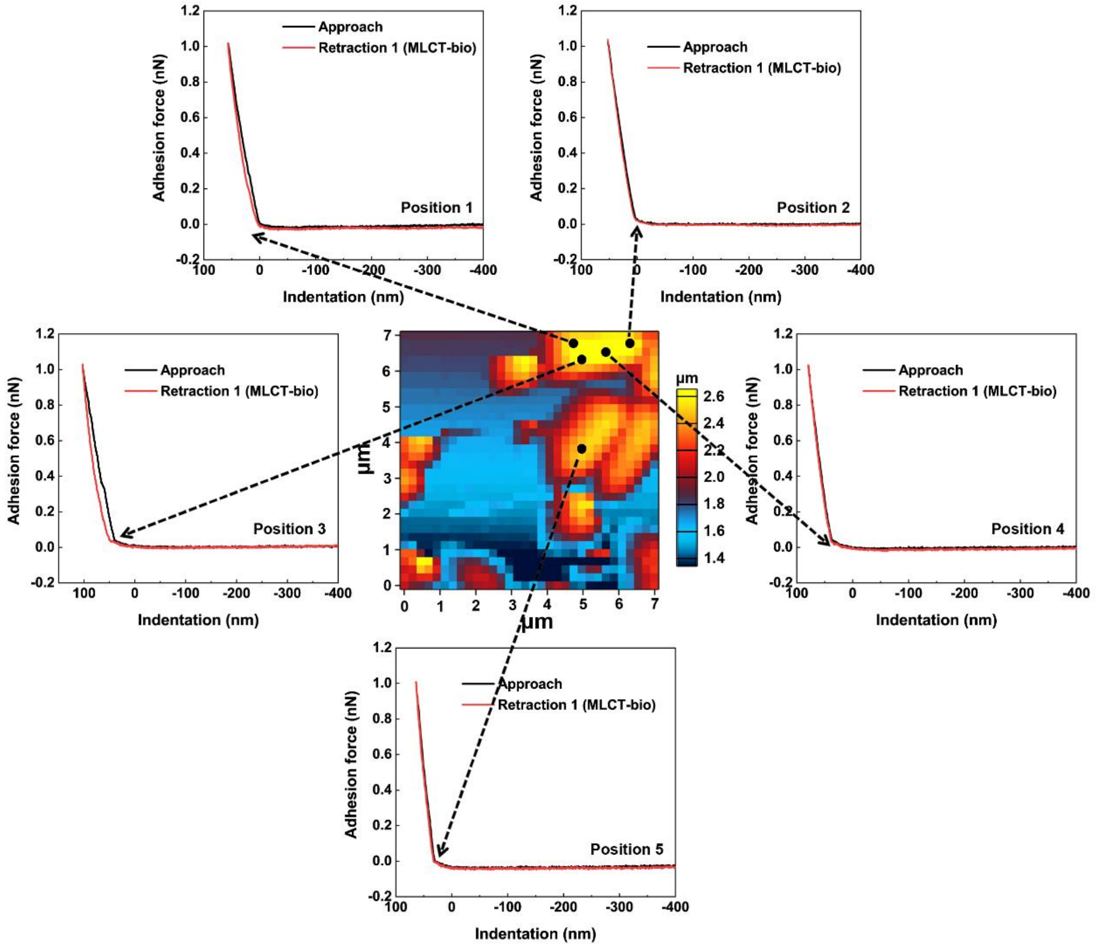

Supplementary Fig. 7 | Adhesion force between an *E. coli* cell and an original AFM tip (positions 1–5). Source data are provided as a Source Data file.

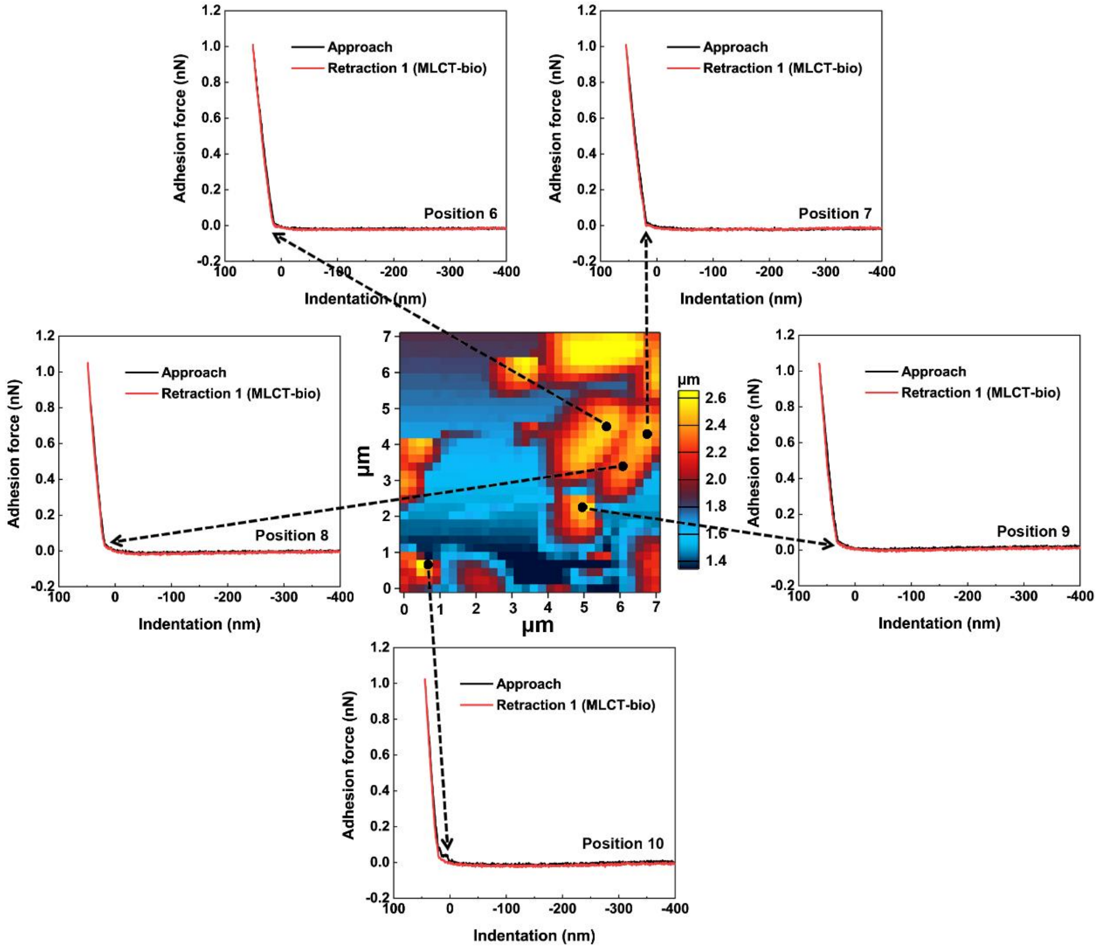

Supplementary Fig. 7 (Continued) | Adhesion force between an *E. coli* cell and an original AFM tip (positions 6–10). Source data are provided as a Source Data file.

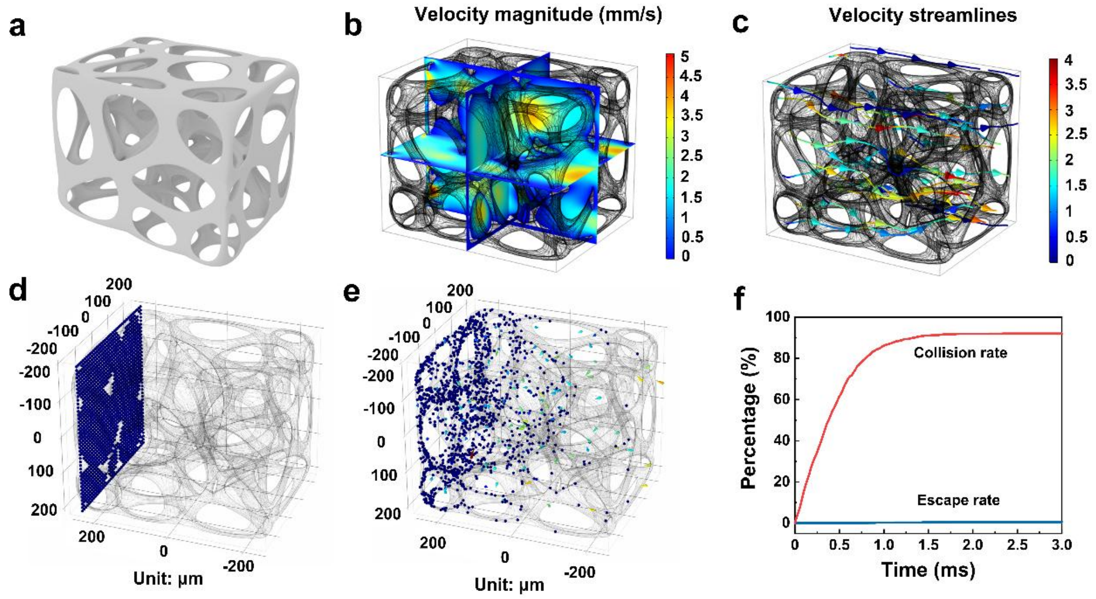

Supplementary Fig. 8 | Simulation of bacterial motion inside a copper foam. a, 3D geometry of the copper foam within a 520 × 420 × 420 μm^3 volume. **b**,**c**, The velocity field inside the copper foam. **d**,**e**, Snapshots of bacterial motion at 0 ms (**d**) and 1 ms (**e**). **f**, The cumulative collision and escape efficiency of 1681 bacteria during a 3-ms simulation. Source data are provided as a Source Data file. In the simulation domain, the total number of bacteria colliding was over 92%. When the thickness of the copper foam in the direction of the water flow exceeds 3 mm (approximately six periodic spaces), the collision rate will be over 99.9999%. In a typical disinfection experiment, the bacteria flowed through two pieces of copper foam with a total thickness of 4 mm. Therefore, we are confident that all the bacteria collided with the surface of the copper foam.

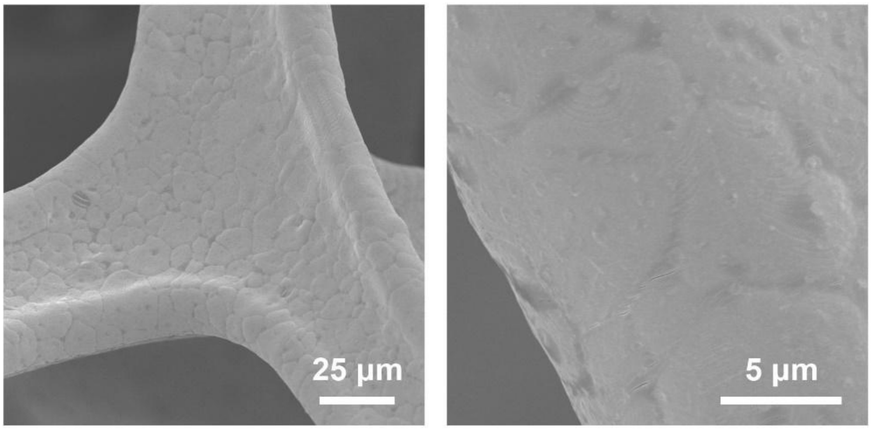

Supplementary Fig. 9 | SEM images of the modified Cu foam. Observations were repeated three times independently with similar results.

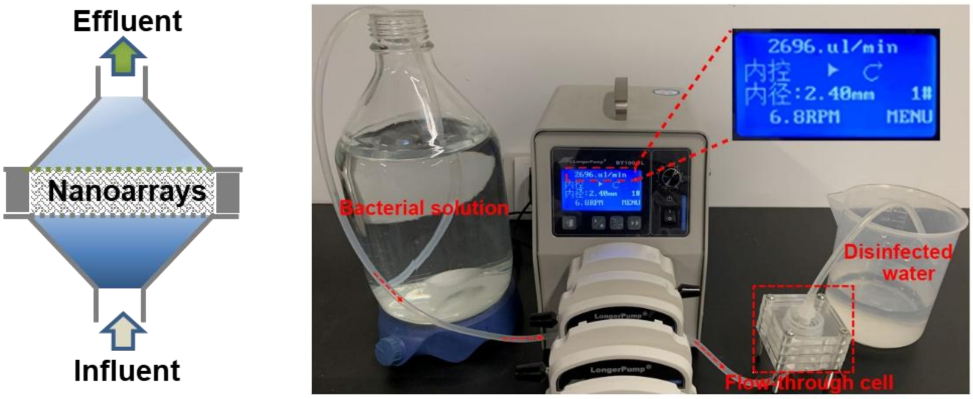

Supplementary Fig. 10 | Schematic of the flow-through disinfection cell and a photographic image of the experimental setup. A peristaltic pump was used to control the flow rate of the bacterial solution at 2.7 mL min^{-1}, corresponding to a flux of about 2 m^3 h^{-1} m^{-2}. The flow-through cell contained two pieces of copper foam.

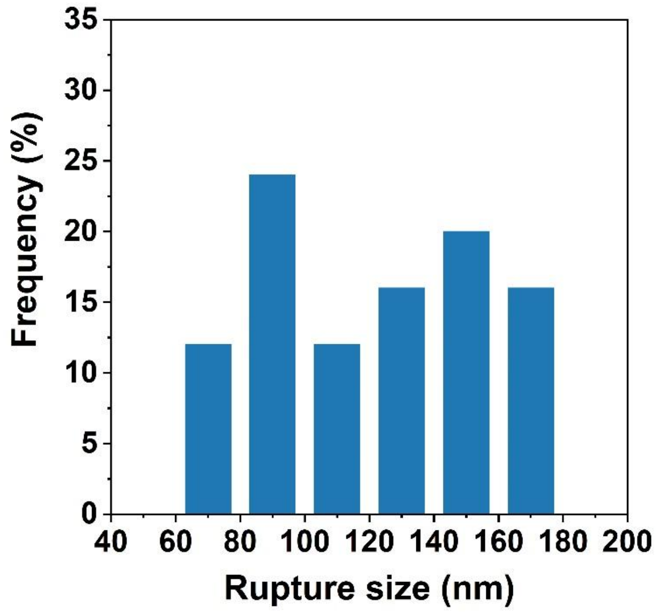

Supplementary Fig. 11 | Distribution of the rupture sizes of the *E. coli* cells treated by the modified NWs. Source data are provided as a Source Data file.

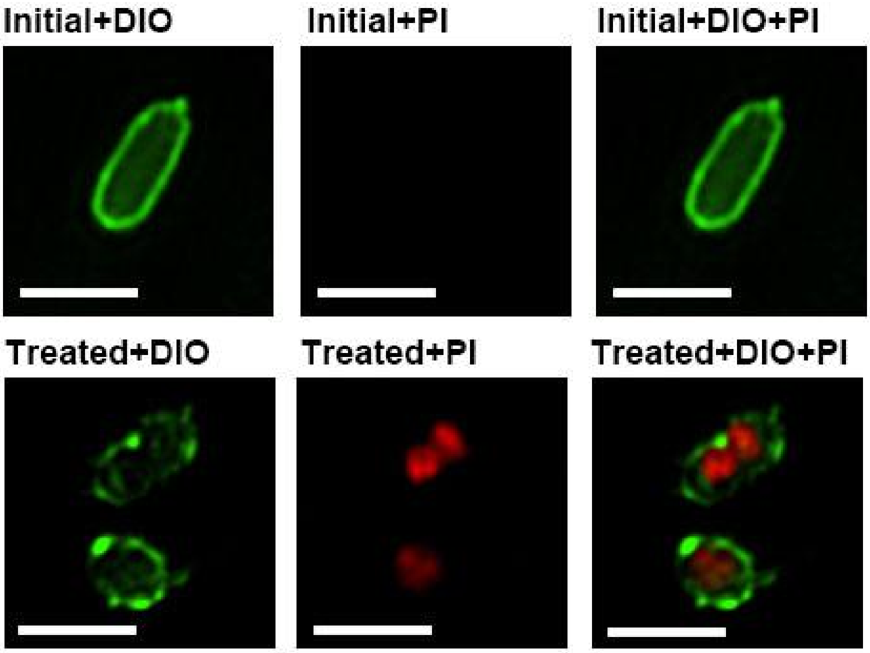

Supplementary Fig. 12 | SIM images of initial *E .coli* and *E .coli* treated with the modified NWs (scale bar = 2 μm). Observations were repeated on eight different cells with similar results.

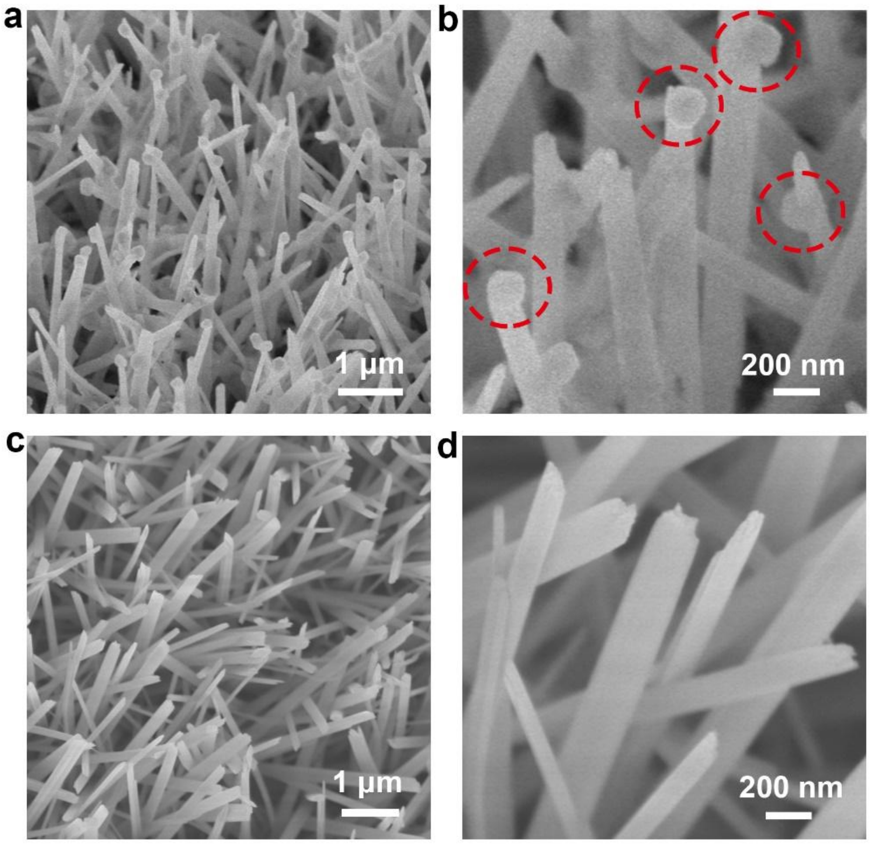

Supplementary Fig. 13 | Morphologies of (**a**,**b**) the modified NWs and (**c**,**d**) the $Cu(OH)_2$ NWs after continous disinfection for over 5 h. Observations were repeated three times independently with similar results.

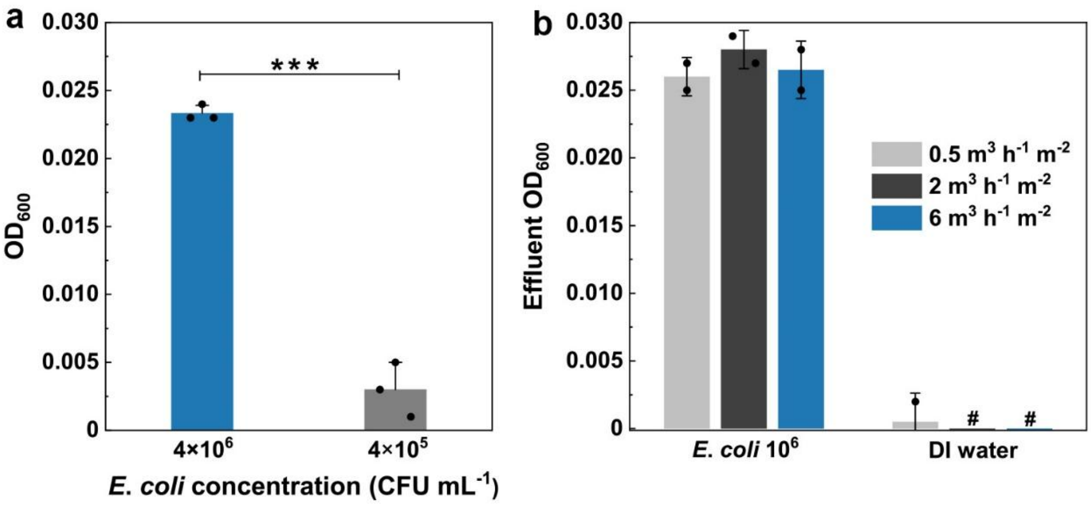

Supplementary Fig. 14 | Optical density measurements. a, OD_{600} of the *E. coli* suspension in different concentrations. *** $p = 0.00007$, which was determined by two-tailed Student's t test. Data are presented as mean ± SD with n = 3 independent measurements. **b**, OD_{600} of the effluent water at different flow rates. The *E. coli* suspension (4×10^6 CFU mL^{-1}, determined by plate count) and the DI water were used as the influent water. Data are presented as mean ± SD with n = 2 independent experiments. # indicates below the detection limit. Source data are provided as a Source Data file.

We first determined the OD_{600} value of the *E. coli* suspension (4×10^6 CFU mL^{-1}, determined by plate count), which lies in 0.02–0.03 at an optical pathlength of 5 cm. When the *E. coli* suspension was diluted tenfold, the OD_{600} value dropped significantly to lower than 0.005 (Supplementary Fig. 14a), confirming that the OD_{600} was sensitive to the change of cell density in water.

The potential influence of released particles from the modified NWs was excluded by using the DI water as the influent. The OD_{600} values of the effluent DI water were near or below the detection limit (Supplementary Fig. 14b), indicating the turbidity of the effluent water was not increased by the material itself. Based on this, we can conclude that the OD_{600} values of the effluent *E. coli* suspension were comparable to the influent, meaning that the density of the bacterial cells was unchanged in the effluent. Thus, the bacteria treated by the modified NWs were not removed by adsorption.

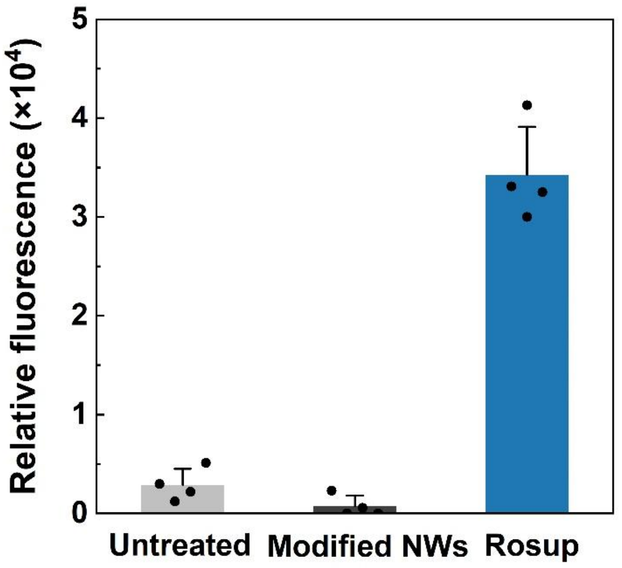

Supplementary Fig. 15 | Measurement of the intracellular ROS in the untreated *E. coli* cells and the *E. coli* cells treated by the modified NWs. The *E. coli* sample treated by Rosup were used as a posive control. Data are presented as mean ± SD with n = 4 independent measurements. Source data are provided as a Source Data file.

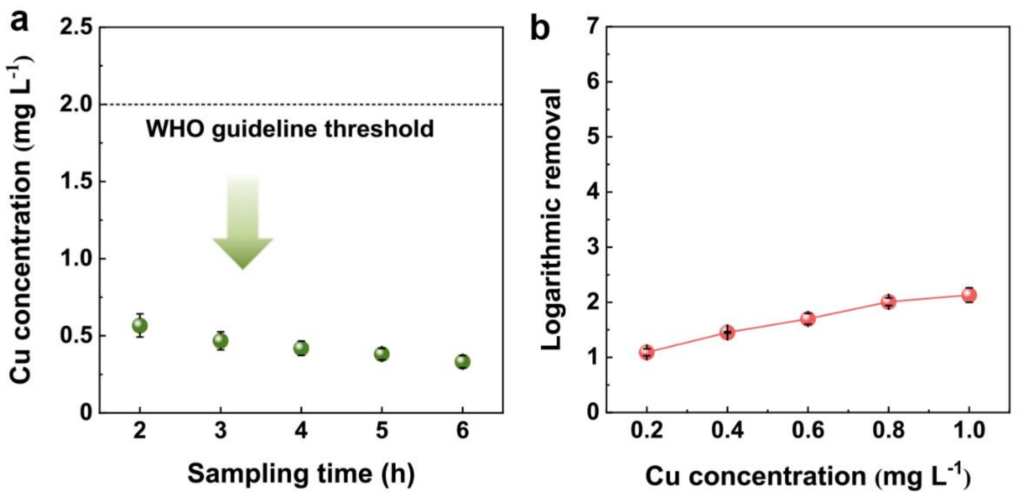

Supplementary Fig. 16 | Impact of released Cu on bacterial inactivation. a, Effluent Cu concentration at different sampling times. **b**, Bactericidal performance of Cu^{2+} against *E. coli*. Data are presented as mean ± SD with n = 3 independent experiments. Source data are provided as a Source Data file.

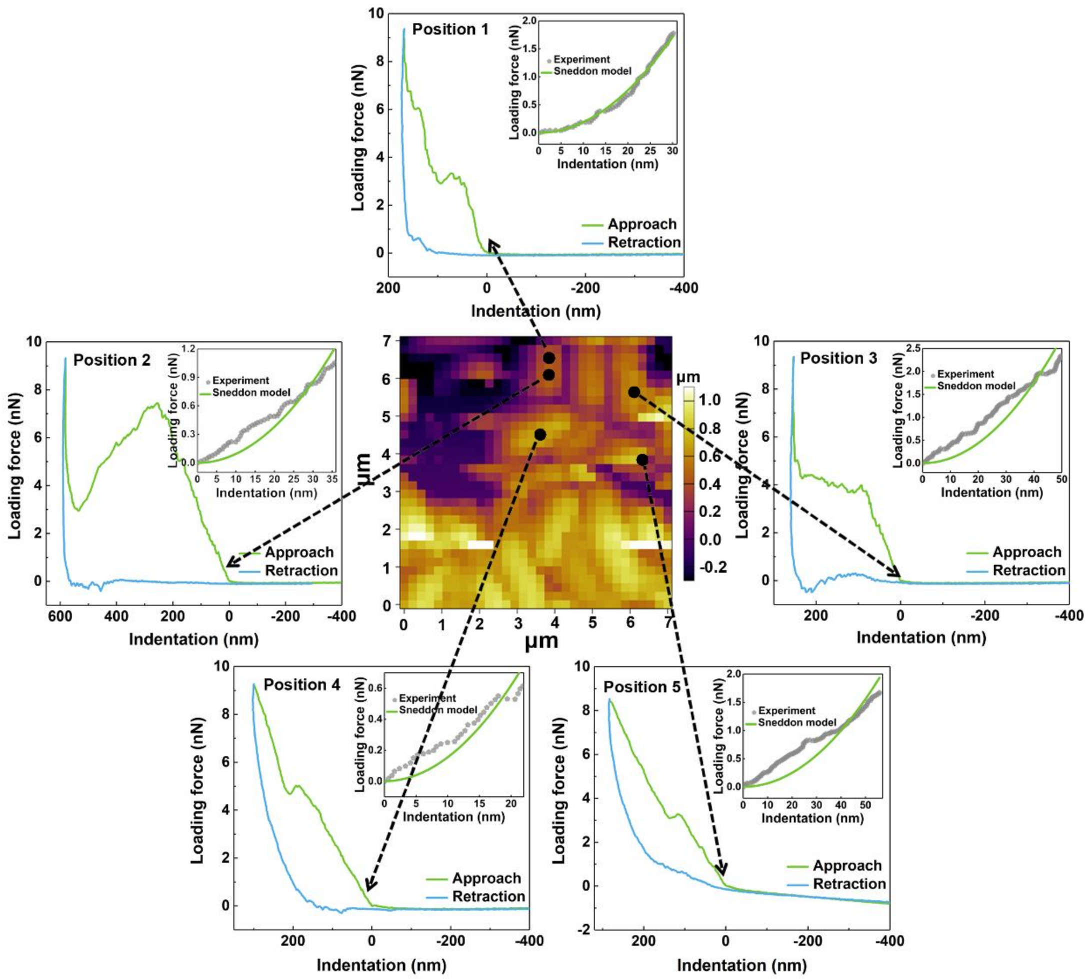

Supplementary Fig. 17 | Puncture curves of *E. coli* (positions 1–5). Source data are provided as a Source Data file.

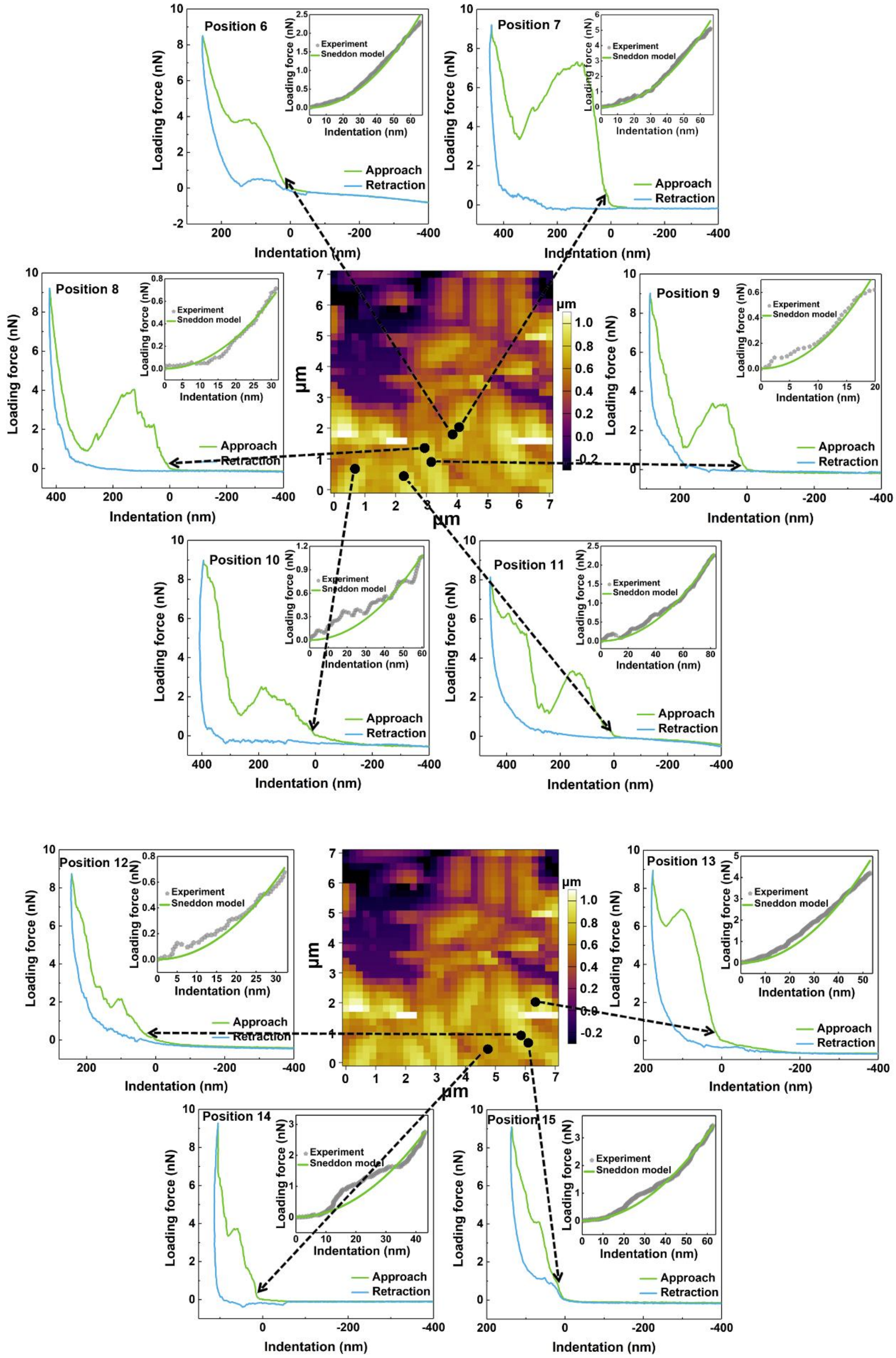

Supplementary Fig. 17 (Continued) | Puncture curves of *E. coli* (positions 6–15). Source data are provided as a Source Data file.

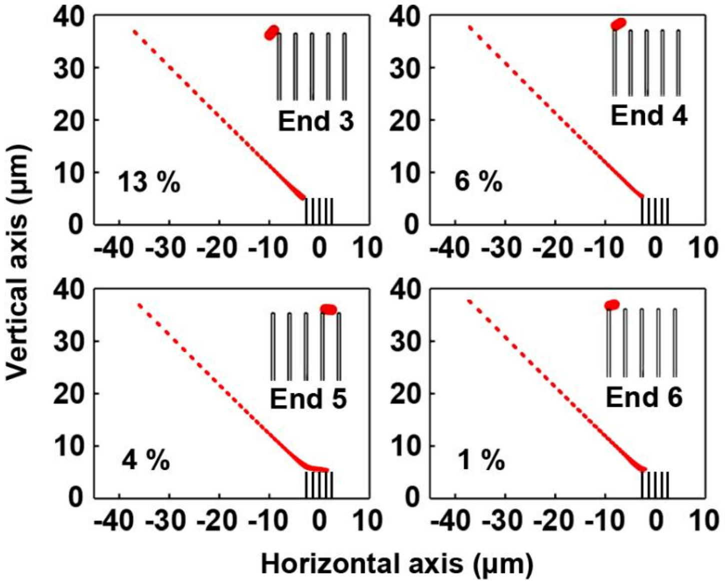

Supplementary Fig. 18 | Four end-contact possibilities between the bacteria and the nanotips.

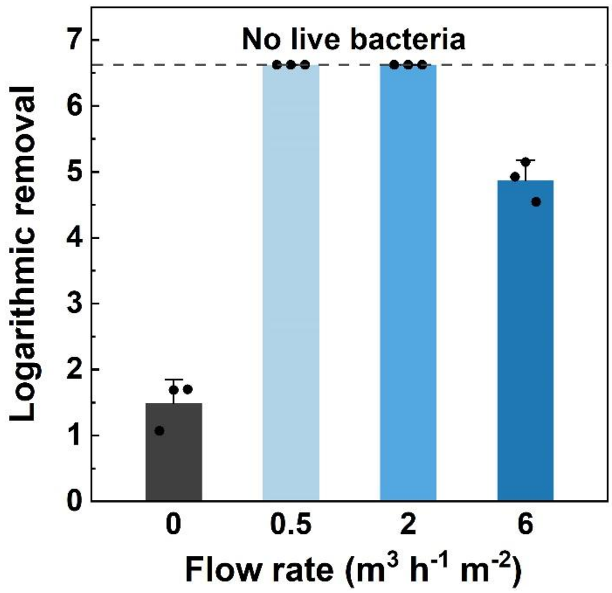

Supplementary Fig. 19 | Influence of different flow rates on the inactivation of bacteria by the modified NWs. The flow rate is given in the form of flux (m^3 h^{-1} m^2). Flow rate of zero represents the bactericidal test of the modified NWs in the static condition. Data are presented as mean ± SD with n = 3 independent experiments. Source data are provided as a Source Data file.

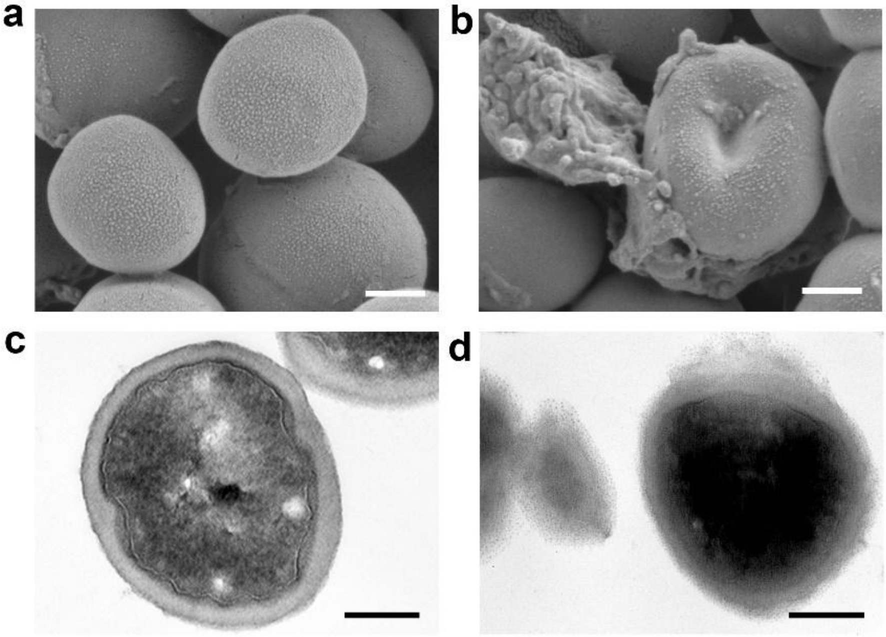

Supplementary Fig. 20 | Morphological change of *S. aureus*. **a**,**b**, SEM images of the initial *S. aureus* (a) and *S. aureus* treated by the modified NWs (b). **c**,**d**, TEM images of the initial *S. aureus* (c) and *S. aureus* treated by modified NWs (d). Scale bar = 200 nm. Observations were repeated three times independently with similar results.

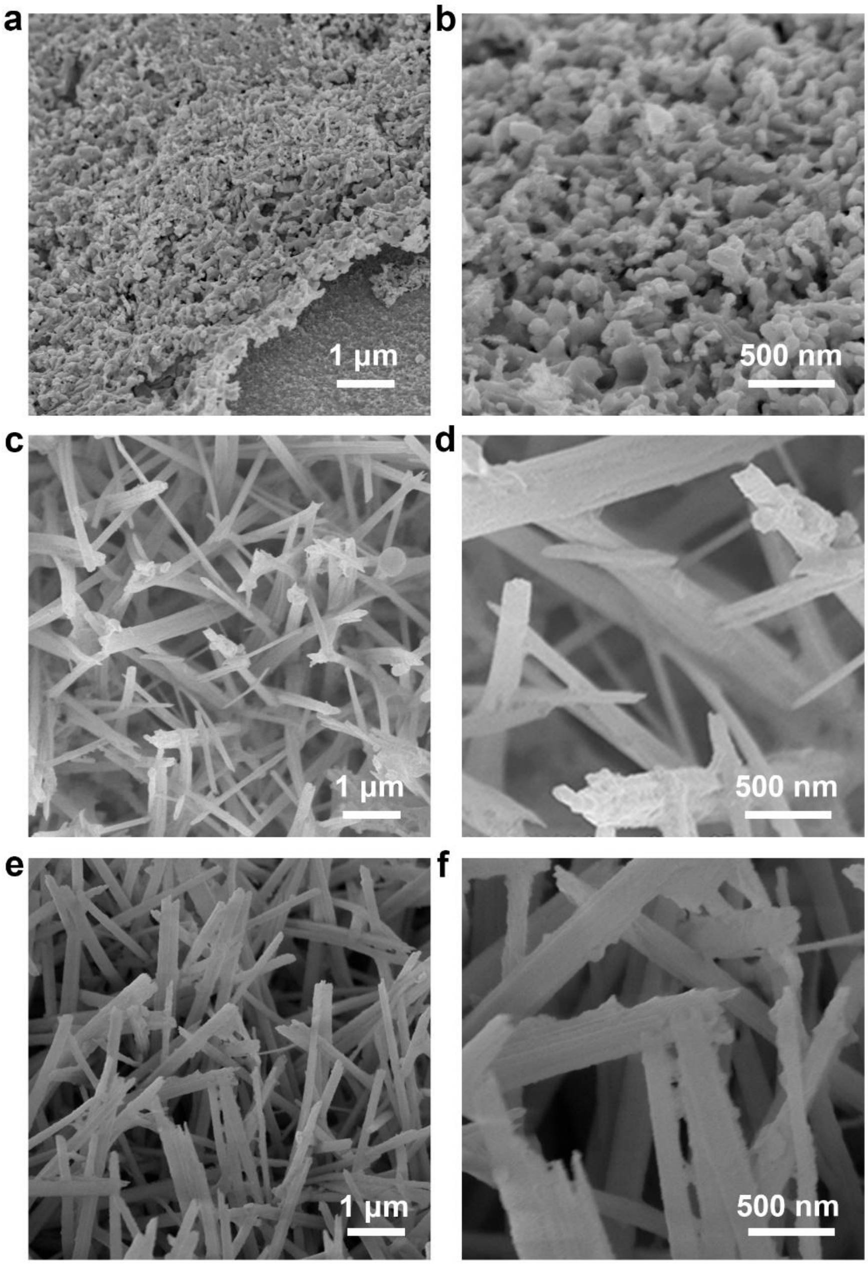

Supplementary Fig. 21 | Morphologies of the modified NWs after 30-day disinfection with a treating capacity of over 10000 V_{water}/V_0. **a**,**b**, Modified NWs in the first unit. **c**,**d**, Modified NWs in the middle unit. **e**,**f**, Modified NWs in the final unit. Observations were repeated three times independently with similar results.

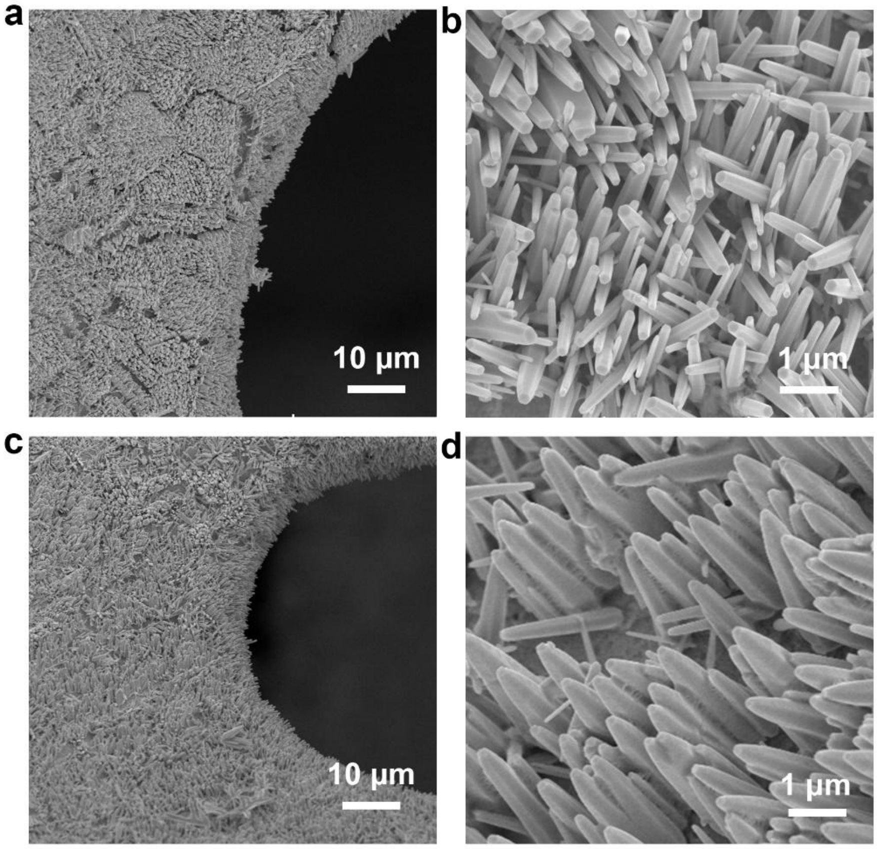

Supplementary Fig. 22 | SEM images of the (**a**,**b**) original and (**c**,**d**) modified ZnO nanorods. Observations were repeated three times independently with similar results.

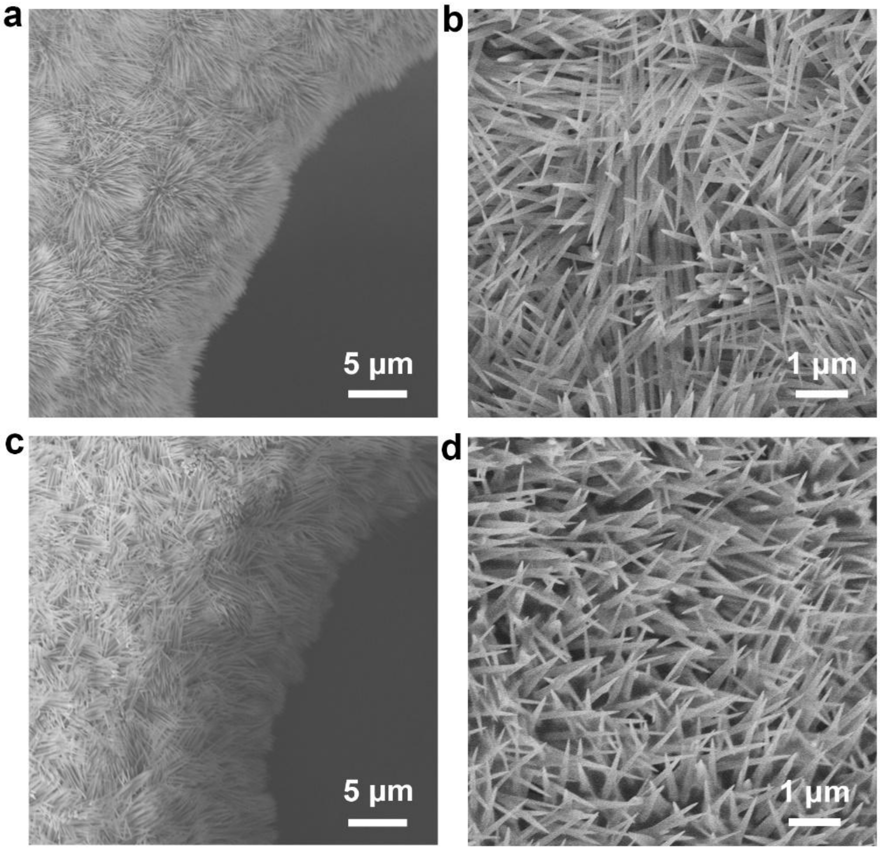

Supplementary Fig. 23 | SEM images of the (**a**,**b**) original and (**c**,**d**) modified Co, Mn-LDH nanoneedles. Observations were repeated three times independently with similar results.

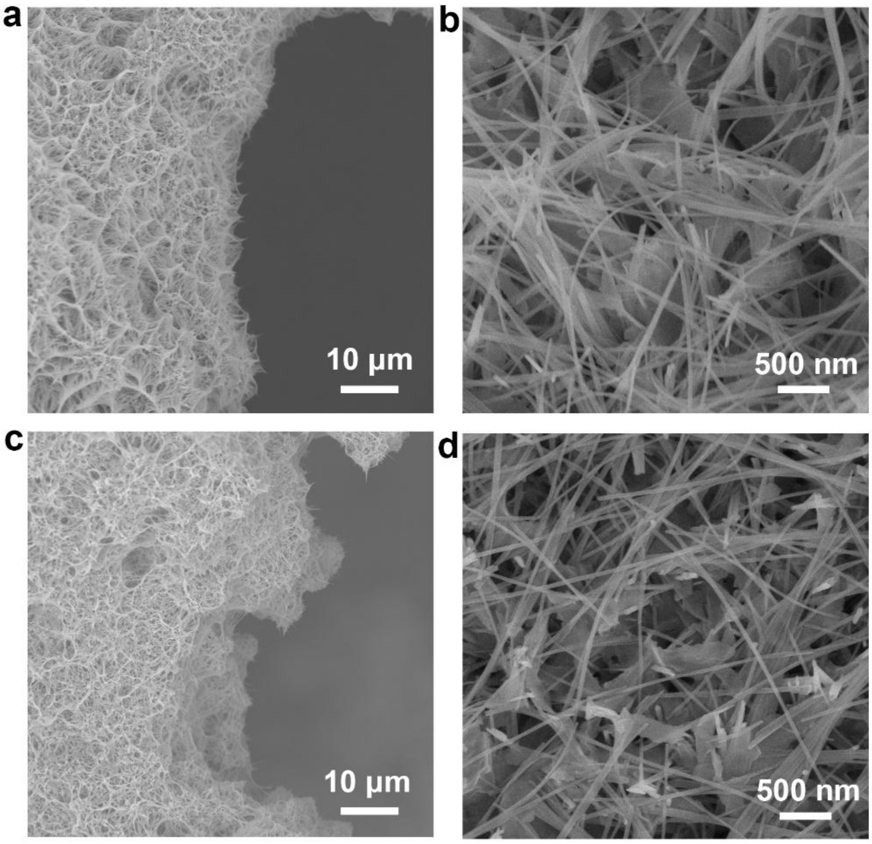

Supplementary Fig. 24 | SEM images of the **(a,b)** original and **(c,d)** modified titanate nanowires. Observations were repeated three times independently with similar results.

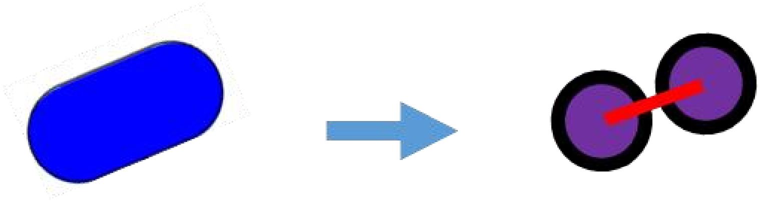

Supplementary Fig. 25 | The bead-stick model of a bacterium.

Supplementary Table 1 | Live bacterial concentrations in the treated and untreated water samples (raw data of Fig. 2f).

Concentration (CFU mL^{-1})	Mean	Std
Influent water	4.5×10^6	2×10^5
Effluent of the modified NWs	0	0
Effluent of the $Cu(OH)_2$ NWs	2.75×10^5	2.5×10^4
Effluent of the modified Cu	4.85×10^5	3.15×10^5

Supplementary Table 2 | Comparison of bactericidal performance with various mechano-bactericidal activities.

Material	Category	Inactivation (log)	Contact time (min)	Bacteria	Initial concentration	Ref.
Black silicon	Nanostructured surface	0.29	180	*P. aeruginosa*	10^6 CFU mL^{-1}	1
PMMA	Nanostructured surface	0.30	1440	*E. coli*	6×10^6 CFU mL^{-1}	2
Black silicon	Nanostructured surface	0.96	1440	*P. aeruginosa*	$OD_{600} = 0.1$	3
Silicon	Nanostructured surface	1.76	360	*E. coli*	10^6 CFU mL^{-1}	4
Titania	Nanostructured surface	0.82	60	*E. coli*	$OD_{600} = 0.3$	5
Titanium	Nanostructured surface	0.28	1080	*P. aeruginosa*	$OD_{600} = 0.3$	6
Black titanium	Nanostructured surface	1.30	240	*E. coli*	$OD_{600} =$ 0.1 ~ 0.15	7
Titanium	Nanostructured surface	0.40	180	*E. coli*	10^6 CFU mL^{-1}	8
Silicon	Nanostructured surface	0.77	180	*E. coli*	$OD_{600} = 0.25$	9
Silicon	Nanostructured surface	1.30	1080	*P. aeruginosa*	$OD_{600} = 0.1$	10
Silicon	Nanostructured surface	0.67	1440	*E. coli*	10^6 CFU mL^{-1}	11
FeOOH	Nanostructured surface	6	1440	*E. coli*	10^6 CFU mL^{-1}	12
Titania	Nanostructured surface	0.22	180	*E. coli*	10^6 CFU mL^{-1}	13
Black silicon	Nanostructured surface	0.48	360	*P. aeruginosa*	10^7 CFU mL^{-1}	14

Zero-valent iron	Metal nanoparticles	3.4	10	*E. coli*	10^6 CFU mL^{-1}	15
Gold	Metal nanoparticles	3	30	*P. aeruginosa*	10^3 CFU mL^{-1}	16
Zero-valent iron	Metal nanoparticles	5.2	60	*E. coli*	10^6 CFU mL^{-1}	17
Silver/ rGO	Metal nanoparticles	1.52	1	*E. coli*	10^3–10^5 CFU mL^{-1}	18
Silver	Metal nanoparticles	3	20	*E. coli*	10^6 CFU mL^{-1}	19
Silver	Metal nanoparticles	2.52	1.81	*E. coli*	10^4–10^6 CFU mL^{-1}	20
Zero-valent iron	Metal nanoparticles	4.2	120	*E. coli*	10^7 CFU mL^{-1}	21
Silver	Metal nanoparticles	3	0.25	*E. coli*	10^8 CFU mL^{-1}	22
Silver/ graphene	Metal nanoparticles	0.63	10	*E. coli*	10^7 CFU mL^{-1}	23
Silver/ chitosan	Metal nanoparticles	3	5	*E. coli*	10^8 CFU mL^{-1}	24
Graphene	Carbon nanomaterials	1.70	600	*E. coli*	8.8×10^5 CFU mL^{-1}	25
Carbon nanotubes	Carbon nanomaterials	0.65	60	*E. coli*	10^7 CFU mL^{-1}	26
Graphene	Carbon nanomaterials	1.30	60	*E. coli*	10^8 CFU mL^{-1}	27
Graphene oxide	Carbon nanomaterials	0.80	60	*E. coli*	10^6 CFU mL^{-1}	28
Graphene oxide	Carbon nanomaterials	0.26	120	*E. coli*	10^6–10^7 CFU mL^{-1}	29

Supplementary Table 2 | (Continued)

Graphene oxide	Carbon nanomaterials	1.70	240	*E. coli*	10^7 CFU mL^{-1}	30
Carbon nanotubes	Carbon nanomaterials	0.91	120	*E. coli*	10^7 CFU mL^{-1}	31
Carbon nanotubes	Carbon nanomaterials	2.15	1080	*P. aeruginosa*	$OD_{600} = 0.1$	32
$Ti_3C_2T_x$	2D materials	1.70	240	*E. coli*	10^7 CFU mL^{-1}	33
MnO_2 /rGO	2D materials	0.09	180	*E. coli*	$OD_{600} = 0.15$	34
$Ti_3C_2T_x$	2D materials	2	1440	*E. coli*	10^4 CFU mL^{-1}	35
MoS_2	2D materials	0.34	60	*E. coli*	10^8 CFU mL^{-1}	36
**Modified NWs**	**Nanostructured surface**	**6.65**	**0.12**	***E. coli***	**10^6–10^7 CFU mL^{-1}**	**This work**

Note: Two representative Gram-negative species *E.* coli and *P. aeruginosa* were selected to evaluate the bactericidal performance of the reported systems. The initial bacterial concentration was in the form of a colony-forming unit per millimeter or substituted by OD_{600}. The inactivation efficiency is defined by $-\log C/C_0$, where C and C_0 represent initial and final bacterial concentration.

Supplementary Table 3 | Values of Young's modulus fitted by the Sneddon and the Hertz model.

No.	Sneddon		Hertz	
	E (MPa)	R^2	*E* (MPa)	R^2
1	0.82	0.94	0.84	0.74
2	0.41	1.54	0.46	1.27
3	0.47	1.65	0.63	1.35
4	0.68	1.67	0.59	1.38
5	0.27	1.70	0.38	1.40
6	0.25	1.13	0.38	0.89
7	0.55	1.10	0.83	0.86
8	0.30	0.90	0.31	0.72
9	0.81	1.30	0.68	1.06
10	0.13	1.67	0.20	1.58
11	0.15	1.17	0.24	1.12
12	0.29	1.38	0.32	1.13
13	0.73	1.21	1.00	1.01
14	0.64	1.37	0.81	1.10
15	0.37	1.13	0.51	0.72
16	0.62	0.91	0.71	0.71
**Average**	**0.46**	—	**0.56**	—

Note: Positions 1–15 are shown in Supplementary Fig. 17 and position 16 is shown in Fig.3a.

Supplementary Table 4 | Young's modulus of various bacteria measured by AFM in previous studies.

Gram	Species	Strain	Condition	*E* (Mpa)	Ref.
–	*Myxococcus xanthus*	DK1622	Liquid	0.25	37
–	*Shewanella putrefaciens*	ATCC BAA-453	Liquid	0.069–0.098	38
–	*Salmonella typhimurium*	Δasd::kan H71-pHC	Liquid	0.4 ± 0.2	39
–	*Escherichia coli*	JM109	Liquid	0.365 ± 0.122	40
–	*Escherichia coli*	TG1	Liquid	1.0 ± 0.3	41
–	*Klebsiella pneumoniae*	AJ218	Liquid	0.006	42
–	*Salmonella*	S.7	Liquid	3.31 ± 0.79	43
–	*Salmonella*	S.12	Liquid	4.29 ± 0.86	43
–	β-Lactam–resistant *Salmonella*	S.44	Liquid	0.58 ± 0.13	43
–	β-Lactam–resistant *Salmonella*	S.79	Liquid	0.40 ± 0.16	43
+	Group B *Streptococcus*	NEM316	Liquid	2.36 ± 0.43	44
+	*Staphylococcus aureus*	ATCC 25923	Liquid	1.764 ±0.218	45
–	*Escherichia coli*	NCTC 9001	Air	221.4 ± 11.9	46
+	*Staphylococcus aureus*	NCTC 8532	Air	95.4	46
+	*Brevibacterium casei*	—	Air	769.28 ± 73.07	47

Note: Most of the reported Young's modulus measured in liquid environment are in the range of 0.1–5 MPa, which are much lower than values measured from air-drying samples, because dehydrating the bacterial cells often causes a drastic increase in cell stiffness. The reported values for *E. coli* cells (0.365–1 MPa) measured in liquid environment are comparable to our measured value (0.5 MPa).

Supplementary Table 5 | Characteristics of the real water samples.

Water sample	pH	Conductivity (μS cm^{-1})	Total organic carbon (mg L^{-1})	Inorganic Carbon (mg L^{-1})
Tap water	7.63	221	2.11	9.02
Reclaimed water	7.50	969	4.64	14.45

Supplementary Table 6 | Geometrical parameters of the nanomaterials used in this study

Nanomaterial	Density /30 μm^2	Length (nm)	Diameter (nm)	Aspect ratio
$Cu(OH)_2$	165	5326 ± 890	199 ± 32	26.6
ZnO	135	2123 ± 539	340 ± 70	6.2
Co, Mn-LDH	195	2883 ± 700	127 ± 20	22.7
Titanate	175	2911 ± 617	47 ± 8	61.9

Note: The nanotip length and diameter, as well as density of the features were assessed using Nano Measurer 1.2. The data were collected from at least 30 individual nanotips in the corresponding SEM images.

Supplementary Table 7 | Composition of the culture media

Type	Ingredient	Concentration (g L^{-1})	pH
Nutrient broth	Peptone	10.0	7.2 ± 0.2
	Beef extract	3.0	
	Sodium chloride	5.0	
Nutrient agar	Peptone	10.0	7.3 ± 0.1
	Beef extract	3.0	
	Sodium chloride	5.0	
	Agar	15.0	

Supplementary References

1 Ivanova, E. P. *et al.* Bactericidal activity of black silicon. *Nat. Commun.* **4**, 2838 (2013).

2 Dickson, M. N. *et al.* Nanopatterned polymer surfaces with bactericidal properties. *Biointerphases* **10**, 021010 (2015).

3 Linklater, D. P. *et al.* Influence of nanoscale topology on bactericidal efficiency of black silicon surfaces. *Nanotechnology* **28**, 245301 (2017).

4 Vassallo, E. *et al.* Bactericidal performance of nanostructured surfaces by fluorocarbon plasma. *Mater. Sci. Eng. C-Mater. Biol. Appl.* **80**, 117-121 (2017).

5 Diu, T. *et al.* Cicada-inspired cell-instructive nanopatterned arrays. *Sci. Rep.* **4**, 1-7 (2014).

6 Bhadra, C. M. *et al.* Antibacterial titanium nano-patterned arrays inspired by dragonfly wings. *Sci. Rep.* **5**, 1-12 (2015).

7 Hasan, J., Jain, S. & Chatterjee, K. Nanoscale topography on black titanium imparts multi-biofunctional properties for orthopedic applications. *Sci. Rep.* **7**, 1-13 (2017).

8 Sengstock, C. *et al.* Structure-related antibacterial activity of a titanium nanostructured surface fabricated by glancing angle sputter deposition. *Nanotechnology* **25**, 195101 (2014).

9 Hasan, J., Raj, S., Yadav, L. & Chatterjee, K. Engineering a nanostructured "super surface" with superhydrophobic and superkilling properties. *RSC Adv.* **5**, 44953-44959 (2015).

10 Ivanova, E. P. *et al.* The multi-faceted mechano-bactericidal mechanism of nanostructured surfaces. *Proc. Natl. Acad. Sci. U.S.A.* **117**, 12598-12605 (2020).

11 Tan, R., Marzolini, N., Jiang, P. & Jang, Y. Bio-inspired polymer thin films with non-close-packed nanopillars for enhanced bactericidal and antireflective properties. *ACS Appl. Polym. Mater.* **2**, 5808-5816 (2020).

12 Yi, G. *et al.* Iron-based nano-structured surfaces with antimicrobial properties. *J. Mat. Chem. B* **8**, 10146-10153 (2020).

13 Jenkins, J. *et al.* Antibacterial effects of nanopillar surfaces are mediated by cell impedance, penetration and induction of oxidative stress. *Nat. Commun.* **11**, 1626 (2020).

14 Zhao, S. *et al.* Programmed death of injured Pseudomonas aeruginosa on mechano-bactericidal surfaces. *Nano Lett.* **22**, 1129-1137 (2022).

15 Lee, C. *et al.* Bactericidal effect of zero-valent iron nanoparticles on Escherichia coli. *Environ. Sci. Technol.* **42**, 4927-4933 (2008).

16 Linklater, D. P. *et al.* Antibacterial action of nanoparticles by lethal stretching of

bacterial cell membranes. *Adv. Mater.* **32**, 2005679 (2020).

17 Li, Z. *et al.* Adsorbed polymer and NOM limits adhesion and toxicity of nano scale zerovalent iron to E. coli. *Environ. Sci. Technol.* **44**, 3462-3467 (2010).

18 Zeng, X., McCarthy, D. T., Deletic, A. & Zhang, X. Silver/reduced graphene oxide hydrogel as novel bactericidal filter for point-of-use water disinfection. *Adv. Funct. Mater.* **25**, 4344-4351 (2015).

19 Zhang, M. *et al.* Magnetically ultraresponsive nanoscavengers for next-generation water purification systems. *Nat. Commun.* **4**, 1866 (2013).

20 Wei, F., Zhao, X., Li, C. & Han, X. A novel strategy for water disinfection with a AgNPs/gelatin sponge filter. *Environ. Sci. Pollut. Res.* **25**, 19480-19487 (2018).

21 Hu, Y. *et al.* Roles of extracellular polymeric substances in the bactericidal effect of nanoscale zero-valent iron: Trade-offs between physical disruption and oxidative damage. *Environ. Sci.: Nano* **6**, 2061-2073 (2019).

22 Loo, S. L. *et al.* Superabsorbent cryogels decorated with silver nanoparticles as a novel water technology for point-of-use disinfection. *Environ. Sci. Technol.* **47**, 9363-9371 (2013).

23 Deng, C. H. *et al.* Preparation of melamine sponge decorated with silver nanoparticles-modified graphene for water disinfection. *J. Colloid Interface Sci.* **488**, 26-38 (2017).

24 Fan, M. *et al.* Facile preparation of silver nanoparticle decorated chitosan cryogels for point-of-use water disinfection. *Sci. Total Environ.* **613**, 1317-1323 (2018).

25 Lu, X. *et al.* Designing melittin-graphene hybrid complexes for enhanced antibacterial activity. *Adv. Healthc. Mater.* **8**, 1801521 (2019).

26 Vecitis, C. D., Zodrow, K. R., Kang, S. & Elimelech, M. Electronic-structure-dependent bacterial cytotoxicity of single-walled carbon nanotubes. *ACS nano* **4**, 5471-5479 (2010).

27 Gollavelli, G., Chang, C. C. & Ling, Y. C. Facile synthesis of smart magnetic graphene for safe drinking water: heavy metal removal and disinfection control. *ACS Sustain. Chem. Eng.* **1**, 462-472 (2013).

28 Akhavan, O. & Ghaderi, E. Toxicity of graphene and graphene oxide nanowalls against bacteria. *ACS nano* **4**, 5731-5736 (2010).

29 Liu, S. *et al.* Lateral dimension-dependent antibacterial activity of graphene oxide sheets. *Langmuir* **28**, 12364-12372 (2012).

30 Zhao, J. *et al.* Graphene oxide-based antibacterial cotton fabrics. *Adv. Healthc. Mater.* **2**, 1259-1266 (2013).

31 Kang, S., Pinault, M., Pfefferle, L. D. & Elimelech, M. Single-walled carbon nanotubes exhibit strong antimicrobial activity. *Langmuir* **23**, 8670-8673 (2007).

32 Linklater, D. P. *et al.* High aspect ratio nanostructures kill bacteria via storage and release of mechanical energy. *ACS nano* **12**, 6657-6667 (2018).

33 Rasool, K. *et al.* Antibacterial activity of $Ti_3C_2T_x$ MXene. *ACS Nano* **10**, 3674-3684 (2016).

34 Alimohammadi, F. *et al.* Antimicrobial properties of 2D MnO_2 and MoS_2 nanomaterials vertically aligned on graphene materials and Ti_3C_2 MXene. *Langmuir* **34**, 7192-7200 (2018).

35 Rasool, K. *et al.* Efficient antibacterial membrane based on two-dimensional $Ti_3C_2T_x$ (MXene) nanosheets. *Sci. Rep.* **7**, 1-11 (2017).

36 Chen, Y. *et al.* Synergetic lipid extraction with oxidative damage amplifies cell-membrane-destructive stresses and enables rapid sterilization. *Angew. Chem. Int. Ed.* **60**, 7744-7751 (2021).

37 Pelling, A. E., Li, Y., Shi, W. & Gimzewski, J. K. Nanoscale visualization and characterization of Myxococcus xanthus cells with atomic force microscopy. *Proc. Natl. Acad. Sci. U.S.A.* **102**, 6484-6489 (2005).

38 Gaboriaud, F. *et al.* Spatially resolved force spectroscopy of bacterial surfaces using force-volume imaging. *Colloids Surf. B. Biointerfaces* **62**, 206-213 (2008).

39 Suo, Z. *et al.* Bacteria survive multiple puncturings of their cell walls. *Langmuir* **25**, 4588-4594 (2009).

40 Chen, Y. Y. *et al.* Surface rigidity change of Escherichia coli after filamentous bacteriophage infection. *Langmuir* **25**, 4607-4614 (2009).

41 Alsteens, D., Trabelsi, H., Soumillion, P. & Dufrêne, Y. F. Multiparametric atomic force microscopy imaging of single bacteriophages extruding from living bacteria. *Nat. Commun.* **4**, 1-7 (2013).

42 Wang, H. *et al.* Nanomechanics measurements of live bacteria reveal a mechanism for bacterial cell protection: The polysaccharide capsule in Klebsiella is a responsive polymer hydrogel that adapts to osmotic stress. *Soft Matter* **9**, 7560-7567 (2013).

43 Liu, L. *et al.* Mechanical penetration of β-lactam–resistant Gram-negative bacteria by programmable nanowires. *Sci. Adv.* **6**, eabb9593 (2020).

44 Saar Dover, R. *et al.* Multiparametric AFM reveals turgor-responsive net-like peptidoglycan architecture in live streptococci. *Nat. Commun.* **6**, 1-10 (2015).

45 Francius, G., Domenech, O., Mingeot-Leclercq, M. P. & Dufrêne, Y. F. Direct observation of Staphylococcus aureus cell wall digestion by lysostaphin. *J. Bacteriol.* **190**, 7904-7909 (2008).

46 Eaton, P. *et al.* Atomic force microscopy study of the antibacterial effects of chitosans on Escherichia coli and Staphylococcus aureus. *Ultramicroscopy* **108**,

1128-1134 (2008).

47 Kumar, U., Vivekanand, K. & Poddar, P. Real-time nanomechanical and topographical mapping on live bacterial cells—Brevibacterium casei under stress due to their exposure to Co^{2+} ions during microbial synthesis of Co_3O_4 nanoparticles. *J. Phys. Chem. B* **113**, 7927-7933 (2009).
