# Supplementary material for: Hydrodynamic tearing of bacteria on nanotips for sustainable water disinfection
Source: Nat Commun. 2023 Sep 15;14:5734. doi: 10.1038/s41467-023-41490-5 (PMC10504294; doi:10.1038/s41467-023-41490-5)
Supplement: Supplementary file 3 — Description of Additional Supplementary Files [file 41467_2023_41490_MOESM3_ESM.pdf]

Description of Additional Supplementary Files:

Supplementary Movie 1: MD simulation of interaction between a POPE membrane and a material with a well depth (ε) value of 0.073 kJ mol-1. The material is shown in green beads, the lipids in the membrane are shown in lines, and the simulation time is 100 ns.

Supplementary Movie 2: MD simulation of interaction between a POPE membrane and a material with a well depth (ε) value of 0.146 kJ mol-1.

Supplementary Movie 3: MD simulation of interaction between a POPE membrane and a material with a well depth (ε) value of 0.256 kJ mol-1.

Supplementary Movie 4: MD simulation of interaction between a POPE membrane and a material with a well depth (ε) value of 0.275 kJ mol-1.

Supplementary Movie 5: MD simulation of interaction between a POPE membrane and a material with a well depth (ε) value of 0.293 kJ mol-1.

Supplementary Movie 6: MD simulation of interaction between a POPE membrane and a material with a well depth (ε) value of 0.586 kJ mol-1.

Supplementary Movie 7: Bacterial motion inside a copper foam. The bacterium was simplified as a spherical particle. The left side of the model was defined as the velocity inlet, while the right side was defined as the outlet. 1681 bacteria were released at the left side of the model and moved.

Supplementary Movie 8: Cell deformation on a nanotip during the collision process by end-contact.

Supplementary Movie 9: Cell deformation on a nanotip during the collision process by middle-contact.

Supplementary Movie 10: Cell deformation on a nanotip during the tearing process by end-contact.

Supplementary Movie 11: Cell deformation on a nanotip during the tearing process by middle-contact.
