# Supplementary material for: Hydrodynamic tearing of bacteria on nanotips for sustainable water disinfection
Source: Nat Commun. 2023 Sep 15;14:5734. doi: 10.1038/s41467-023-41490-5 (PMC10504294; doi:10.1038/s41467-023-41490-5)
Supplement: Supplementary file 15 — Reporting Summary [file 41467_2023_41490_MOESM15_ESM.pdf]

nature portfolio

Corresponding author(s): Hong-Ying Hu; Cheng Yang; Qianyuan Wu

Last updated by author(s): Aug 16, 2023

Reporting Summary

Nature Portfolio wishes to improve the reproducibility of the work that we publish. This form provides structure for consistency and transparency in reporting. For further information on Nature Portfolio policies, see our Editorial Policies and the Editorial Policy Checklist.

Statistics

For all statistical analyses, confirm that the following items are present in the figure legend, table legend, main text, or Methods section.

n/a	Confirmed	
☐	☒	The exact sample size (*n*) for each experimental group/condition, given as a discrete number and unit of measurement
☐	☒	A statement on whether measurements were taken from distinct samples or whether the same sample was measured repeatedly
☐	☒	The statistical test(s) used AND whether they are one- or two-sided<br>*Only common tests should be described solely by name; describe more complex techniques in the Methods section.*
☒	☐	A description of all covariates tested
☒	☐	A description of any assumptions or corrections, such as tests of normality and adjustment for multiple comparisons
☐	☒	A full description of the statistical parameters including central tendency (e.g. means) or other basic estimates (e.g. regression coefficient) AND variation (e.g. standard deviation) or associated estimates of uncertainty (e.g. confidence intervals)
☐	☒	For null hypothesis testing, the test statistic (e.g. *F*, *t*, *r*) with confidence intervals, effect sizes, degrees of freedom and *P* value noted<br>*Give P values as exact values whenever suitable.*
☒	☐	For Bayesian analysis, information on the choice of priors and Markov chain Monte Carlo settings
☒	☐	For hierarchical and complex designs, identification of the appropriate level for tests and full reporting of outcomes
☒	☐	Estimates of effect sizes (e.g. Cohen's *d*, Pearson's *r*), indicating how they were calculated

Our web collection on statistics for biologists contains articles on many of the points above.

Software and code

Policy information about availability of computer code

Data collection	No software was used
Data analysis	Parameters of sp2 carbon atoms based on the INTERFACE force field were used. CHARMM36m all-atom force field parameters for 1-palmitoyl-2-oleoyl-sn-glycero-3-phosphoethanolamine (POPE) lipids, water model of transferable intermolecular potential 3P and ions (Na+, Cl-) were used. CHARMM-GUI webserver and GROMACS tools were used to set up all simulation systems, which were performed using GROMACS software (version 2019.4). Bacterial motion inside a porous copper foam was simulated by the commercial software package ANSYS Fluent (2020 R2). The finite element method was used to explore the bacterial deformation, which was conducted using the commercial software ABAQUS 6.11. The geometrical parameters of the nanomaterials were assessed using Nano Measurer 1.2.

For manuscripts utilizing custom algorithms or software that are central to the research but not yet described in published literature, software must be made available to editors and reviewers. We strongly encourage code deposition in a community repository (e.g. GitHub). See the Nature Portfolio guidelines for submitting code & software for further information.

Data

Policy information about availability of data

All manuscripts must include a data availability statement. This statement should provide the following information, where applicable:

- Accession codes, unique identifiers, or web links for publicly available datasets
- A description of any restrictions on data availability
- For clinical datasets or third party data, please ensure that the statement adheres to our policy

All data are available in the main text and the supplementary information. Source data are provided with this paper.

Research involving human participants, their data, or biological material

Policy information about studies with human participants or human data. See also policy information about sex, gender (identity/presentation), and sexual orientation and race, ethnicity and racism.

Reporting on sex and gender	This is not relevant to our study.
Reporting on race, ethnicity, or other socially relevant groupings	This is not relevant to our study.
Population characteristics	This is not relevant to our study.
Recruitment	This is not relevant to our study.
Ethics oversight	This is not relevant to our study.

Note that full information on the approval of the study protocol must also be provided in the manuscript.

Field-specific reporting

Please select the one below that is the best fit for your research. If you are not sure, read the appropriate sections before making your selection.

☐ Life sciences ☐ Behavioural & social sciences ☒ Ecological, evolutionary & environmental sciences

For a reference copy of the document with all sections, see nature.com/documents/nr-reporting-summary-flat.pdf

Ecological, evolutionary & environmental sciences study design

All studies must disclose on these points even when the disclosure is negative.

Study description	The bactericidal tests were completed within one hour, and about 160-mL water was treated for each test. For each type of material, three sets of flow-through cells were set up and operated independently. The bactericidal efficiency for each type of material was counted based on the three replicates.
Research sample	Three representative Gram-negative and Gram-positive bacteria were used in this study, including Escherichia coli (CGMCC 1.3373), Pseudomonas aeruginosa (CGMCC 1.12483), and Staphylococcus aureus (CGMCC 1.12409), which were frequently detected in municipal wastewater and polluted surface water. Each type of bacteria was cultured at 37 ℃ with shaking at 150 rpm for 12 h, washed to remove the nutrient medium and diluted with DI water to obtain a bacterial suspension of 10^6–10^7 CFU mL-1.
Sampling strategy	The live bacterial concentrations in the influent and effluent water were determined by a standard plate count method. Specifically, 1 mL of each water sample was first collected in a sterile centrifuge tube, which was sufficient for diluting and spreading on three plates. 100 μL of the sample was added to another sterile centrifuge tube containing 900 μL sterile DI water to dilute tenfold. This was repeated four times to prepare serial dilutions (1:10, 1:100, 1:1000 and 1:10000). Once diluted, 100 μL of the dilution sample from various dilutions was spread onto a sterile Petri plate (in triplicate for each dilution) to which molten and cooled nutrient agar medium was added. Following 24-h incubation at 37 ℃, the number of the bacterial colonies developed on the plates can be counted, and the concentration of bacteria in the original water sample was obtained by multiplying the number of colonies obtained per plate by the dilution factor.
Data collection	For each type of material, three sets of flow-through cells were set up and operated independently. The bactericidal efficiency for each type of material was counted based on the three replicates. After the disinfection test, the live bacterial concentrations in the influent and effluent water were measured by Lu Peng using a standard plate count method.
Timing and spatial scale	The bactericidal tests for different nanomaterials was completed within one hour and the bacterial concentrations in the influent and effluent water were determined immediately after the test. Therefore, we do not need to collect data over a time range or spatial scale.<br>The 30-d continuous disinfection was conducted from 2021. 3.16 to 2021. 4. 17.

Data exclusions	No data were excluded from the analyses.
Reproducibility	We have tested the disinfection performance of the carbon-coated Cu(OH)2 nanowires for at least three times, and confirmed the disinfection performance of the carbon-coated nanowires was largely increased compared with the control materials, including the original Cu(OH)2 nanowires and the carbon coated Cu foam. This results have been reproduced by two independent colleagues in the lab.
Randomization	This study aims at disinfection of bacteria in water. This is not relevant to our study.
Blinding	This study aims at disinfection of bacteria in water. Therefore, blinding is not relevant to our study.

Did the study involve field work? ☐ Yes ☒ No

Reporting for specific materials, systems and methods

We require information from authors about some types of materials, experimental systems and methods used in many studies. Here, indicate whether each material, system or method listed is relevant to your study. If you are not sure if a list item applies to your research, read the appropriate section before selecting a response.

Materials & experimental systems

n/a	Involved in the study
☒	☐ Antibodies
☒	☐ Eukaryotic cell lines
☒	☐ Palaeontology and archaeology
☒	☐ Animals and other organisms
☒	☐ Clinical data
☒	☐ Dual use research of concern
☒	☐ Plants

Methods

n/a	Involved in the study
☒	☐ ChIP-seq
☒	☐ Flow cytometry
☒	☐ MRI-based neuroimaging
